# Effect of transdermal drug delivery patches on the stratum corneum: *in vivo* inspection with a handheld terahertz probe

**DOI:** 10.1364/BOE.513557

**Published:** 2024-04-16

**Authors:** Arthur I. Hernandez-Serrano, Xuefei Ding, Goncalo Costa, Gabit Nurumbetov, David M. Haddleton, Emma Pickwell-MacPherson

**Affiliations:** 1 Department of Physics, University of Warwick, Gibbet Hill Road, Coventry CV4 7AL, UK; 2 Medherant Ltd., The Venture Centre, University of Warwick Science Park, Coventry CV4 7EZ, UK; 3 Department of Chemistry, University of Warwick, Gibbet Hill Road, Coventry CV4 7AL, UK

## Abstract

Transdermal drug delivery patches are a good alternative to hypodermic drug injection. The drug delivery efficiency depends strongly on the hydration of the skin under treatment, and therefore, it is essential to study the effects on the skin induced by the application of these medical-grade patches. Terahertz (THz) spectroscopy shows great promise for non-invasive skin evaluation due to its high sensitivity to subtle changes in water content, low power and non-ionizing properties. In this work, we study the effects of transdermal drug delivery patches (three fully occlusive and three partially occlusive) applied on the upper arms of ten volunteers for a maximum period of 28 h. Three different levels of propylene glycol (0 %, 3 % and 6 %) are added to the patches as excipient. By performing multilayer analysis, we successfully retrieve the water content of the stratum corneum (SC) which is the outermost layer of skin, as well as its thickness at different times before and after applying the patches. This study demonstrates the potential of using THz sensing for non invasive skin monitoring and has wide applications for skin evaluation as well as the development of skin products.

## 1. Introduction

Transdermal drug delivery (TDD) patches are becoming increasingly popular for applying medical treatments since they can be self-administered and are non-invasive [1,2]. TDD patches also have the capability of preventing over-dose situations by delivering a limited amount of drug at a relatively stable rate. However, studies have reported that skin hydration level has major impact on the penetration of drug and thus affect the delivery rate [3]. The skin is composed of three main layers, namely (from the outermost to the innermost): the epidermis; dermis; and subcutaneous tissue. The stratum corneum (SC) is the outermost layer of the epidermis and serves as a barrier to control exchange of materials for both directions; the increase in skin hydration will result in SC swelling and reduce the barrier function [4]. Note that there is a water concentration gradient across the SC, but the water concentration of the epidermis beneath it has been found to be fairly constant. Here-on, for clarity, we refer to the remaining epidermis beneath the SC as the epidermis. To improve the consistency in drug delivery rate for TDD patches, it is necessary to study how TDD patches affect the water content in the SC without active ingredients involved. In this case, the backing material and excipient composition in the patch are the main variables to consider. Backing materials with different occlusive features have varied impact on skin’s response to patches: a fully occlusive backing prevents molecular exchange with the environment which is useful when the patch contains toxic drugs [5], whereas a partially occlusive backing allows the skin to breathe avoiding skin irritation due to over-accumulation of water [6]. The excipient propylene glycol (PG) is included in TDD patches to enhance the permeation of drug and it is commonly added at several concentration levels, which may have an effect on the skin hydration [7,8]. All current methods to evaluate TDD patches are based on the measurement of transepidermal water loss [9] which is not accurate and has no robust indication of permeability through the SC.

Terahertz (THz) radiation is non-ionizing and very sensitive to changes in water content due to strong hydrogen bond absorptions in the THz region. This has motivated research into utilizing THz sensing for various biomedical applications including the diagnosis of skin cancer [10], evaluation of burn wounds [11], sensing the SC hydration profile [12–14] and monitoring transdermal drug delivery processes [15–19]. As a label-free, non-destructive sensing method for TDD, Kim et al. conducted *ex vivo* THz imaging to visualize the penetration of topical drug on excised mouse skin [15] and Wang et al. compared the efficacy of different TDD methods with similar THz techniques [16]. In a previous study we found that the corneometer is unreliable at measuring skin hydration [20]. We have shown how we can use THz measurements to give a quantitative measure of skin thickness and hydration by fitting a numerical stratified skin model to the experimental data from over 300 volunteers [21]. Additionally, *in vivo* THz measurements on human skin treated with TDD patches have been performed by our group: Lindley-Hatcher *et al.* demonstrated the potential of using *in vivo* THz sensing to quantify the response of skin to the application of patches with different backing materials [20]. They conducted single point measurements in a pilot study to serve as a proof of concept. Later on, Ding *et al.* increased the experimental scale and analyzed the response of skin to the patches by classifying the volunteers into groups according to their original state of skin hydration [19]. Barker et al. [22] used sparse deconvolution to show that it is even possible to probe the skin beneath the patch whilst the patch is still in place. However, the aforementioned TDD studies quantified skin hydration in an indirect way by analyzing variables such as the peak-to-peak amplitude of reflected waveform, but didn’t apply numerical skin models to directly determine the hydration profile and thickness change in the SC. Another issue in these first studies was that the patches often came off before all the scheduled measurements could be made due to placement on the forearm where they can be easily dislodged.

In this work, we use a handheld THz-TDS reflection system to measure patches on the upper arm. This is, in practice, the most common area to apply TDD patches and is less prone to accidental removal. More details about the handheld probe and its robustness and flexibility are given in Ref. [23]. We study patches with two different backings and three concentration levels of propylene glycol on ten subjects for a duration of 28hrs. We use multilayer models (with two skin layers) combined with effective medium theory to extract the SC water content and thickness as well as the water gradient distribution within the SC and part of epidermis.

## 2. Materials and experimental setup

### 2.1 Measurement protocol

**Fig. 1.**
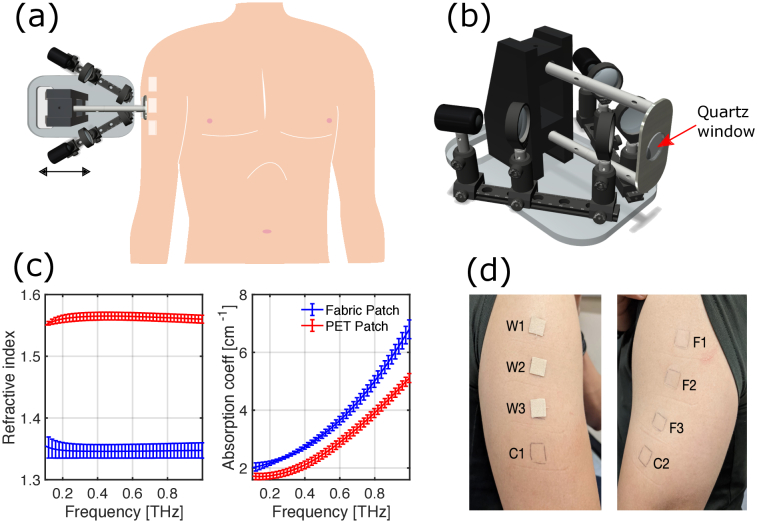
(a) A schematic diagram of the experimental procedure. (b) A diagram of the handheld system. (c) Measured refractive index and absorption coefficient of the film and woven patches. (d) A photo of the left and right upper arms of a subject with the film/woven patches on. C1 and C2 indicate the control areas.

The study was approved by the Biomedical and Scientific Research Ethics Committee at Warwick University (Application number BSREC REGO-2018-2273 AM04). In the following experiments, we used six different types of patches with backing materials of polyethylene terephthalate (PET) and woven material. All patches were the same size with dimensions 1.2 cm x 1.2 cm. The PET patches are fully occlusive while the woven patches are partially occlusive. The patches do not contain active ingredients (drugs) as here we are interested in the performance of the patch itself on the skin. Propylene glycol (PG) with concentration levels of 0 %, 3 % and 6 % is included in the patches as excipient, which is commonly used to enhance the penetration of drugs through the skin [19]. Three PET patches with different excipient levels were applied on the left upper arm of ten healthy volunteers while three woven patches were applied to the right arm as shown in Fig. 1 (a) and (d). In Fig. 1 (d) the PET patches are identified with the letter *F* (film) and woven patches with the letter *W*. In the same figure, two additional regions marked as C1 and C2 are untreated areas serving as the control regions to compare the effects of the patches and account for the natural variation of skin. These eight regions were measured with the THz handheld probe before and after applying the patches for 24hrs.

Blocking the skin’s pores causes water to accumulate in the SC, which we refer to as the "occlusion process". We can monitor this progressive change in water content of the skin through the associated changes in THz reflectivity. The resulting occlusion curve depends on the porosity of the material used to block the skin, the contact pressure and the elapsed occlusion time. We controlled these variables carefully throughout the investigation to make meaningful comparisons. Wearing the patches occludes the skin and causes water to accumulate over the 24hr period. We investigate this effect by measuring the THz response immediately after patch removal for 1 minute (with the skin in contact with the quartz window of the THz probe) – the resulting occlusion curve enables us to determine the skin hydration level. To control for other external variables such as if the volunteer has exercised or drunk water, we also measure a control region of skin on each arm such that a relative change comparison can be made as detailed in Ref. [24].

### 2.2 THz handheld probe

We customised the Terasmart spectrometer from Menlo [25] to be in a handheld configuration for flexible and robust *in vivo* operation. The TeraSmart is a fiber coupled system. By using a pair of photoconductive antennas, the system is able to emit and detect a train of broadband electromagnetic pulses with a time duration of 1 ps with a usable bandwidth of up to 0.1-5 THz with a dynamic range of 90 dB. Fig. 1 (b) shows a schematic diagram of the handheld system: it consists of a reflection setup in which s-polarized THz light is incident on a quartz window at 30 degrees [18]. This quartz window is in contact with skin and the THz light reflected from the quartz-skin interface is analyzed. The thickness and refractive index of the quartz window affect the focal position and equations used in the analysis. We used a quartz window with a thickness of 2 mm and a refractive index of 1.95−0.0048i [26] for all our measurements in this study. Force sensitive resistors, as detailed in [23] were used to keep constant contact pressure. For this work, a measurement comprising four pulses per second for one minute was conducted for each region on the upper arm of the volunteers, during which time the quartz window of the handheld system remained in contact with the skin. The usable bandwidth in our study was 0.1-1 THz due to the high attenuation of THz light by the skin: the higher frequency components reflected are beneath the noise floor.

To study the interaction of the patches with skin, we characterized their THz dielectric properties and used numerical modelling methods (see Section 3.) to determine the hydration and thickness of the SC.

### 2.3 Patch characterisation

All six patches were measured in a THz transmission configuration with the same set up as detailed in Ref. [20]. In Fig. 1 (c) the refractive index and absorption coefficients for both PET and woven patches are shown. During the measurements, it was clear that the refractive index of the patches did not change significantly for different excipient concentrations. The curves shown in Fig. 1 (c) are the mean values of the refractive indices and absorption coefficients of three measurements while the error bars correspond to the standard deviation. From this figure it is evident that the refractive index of the woven patches is lower than the PET (or film) patches. This is because the woven material is highly porous and contains air which reduces the effective refractive index of the patch, while the PET patches are made from a uniform and homogeneous material. Additionally, the porous structure increases the absorption coefficient values for the woven patches relative to the film patches due to scattering losses.

## 3. Numerical modelling

**Fig. 2.**
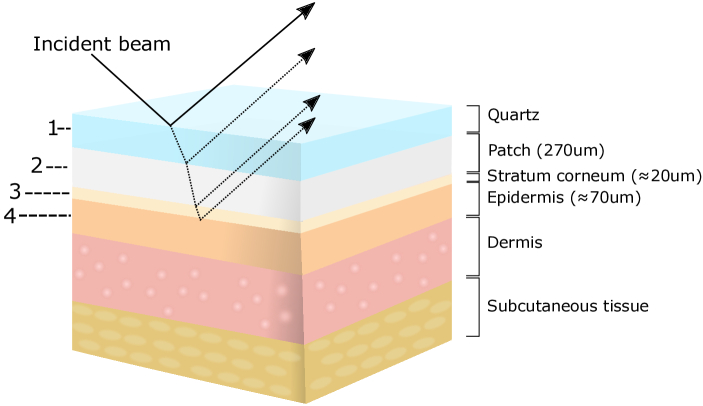
A diagram showing the multilayer structure of skin. When measured with the patch in place, the structure consists of four layers: quartz, patch, SC and epidermis. For the control measurements or measurements after removal of the patch, the structure changes to a tri-layer system.

The skin, patch and quartz probe window can be modelled as a multilayer structure as shown in Fig. 2, consisting of either three or four layers: quartz, patch (when present), SC and epidermis. Due to the high absorption coefficient of skin in the THz range, the THz light cannot penetrate deeply enough to probe skin layers beneath the epidermis such as the dermis or subcutaneous tissue [27], and reflections from these lower layers can be neglected. As shown in Fig. 2, an incident beam will experience multiple reflection after interacting with the sample, and the superposition of these reflections is received in the detector.

### 3.1 M1: the tri-layer model

Equation (1) can be used to model a tri-layer system, i.e. the control areas or treated areas after peeling off the patches (quartz-sc-epidermis) [28]:

(1) $$r_{234,s}=\frac{r_{23,s}+r_{34,s}exp(-2i\beta )}{1+r_{23,s}r_{34,s}exp(-2i\beta )},$$

in which $r_{234,s}$ is the complex reflection coefficient from the layers 2,3 and 4 shown in Fig. 2 for *s* polarization; $r_{ij,s}$ stands for the Fresnel coefficient of reflection in the interface *ij* for *s* polarization; $\beta =k_{0}dn_{sc}cos\theta_{sc}$ , where $k_{0}$ is the propagation constant in vacuum, ${\text{n}}_{sc}$ is the refractive index of the SC (layer 3 in this example), ${\text{d}}_{sc}$ is thickness and $\theta_{sc}$ is the incident angle. The refractive index and the absorption coefficient of the quartz window are determined from transmission measurement and used in the Fresnel coefficients. The incident angle of the THz beam in air is known (30 degrees) and is used to determine $\theta_{sc}$ through Snell’s law.

### 3.2 M2: the four-layer model

In order to adapt the tri-layer model for a four-layer system (quartz-patch-sc-epidermis), Eq. (1) has to be employed in a recursive fashion [28],

(2) $$r_{1234,s}=\frac{r_{12,s}+r_{234,s}exp(-2i\beta_{1})}{1+r_{12,s}r_{234,s}exp(-2i\beta_{1})},$$

in which $r_{234,s}$ is given by eq. (1), $\beta_{1}=k_{0}d_{p}n_{p}cos\theta_{p}$ and $d_{p}$ , $n_{p}$ and $\theta_{p}$ are the thickness, refractive index and incident angle of the patch, respectively. The patch properties are also measured in transmission and are fed into the equations so that the properties of the SC can be extracted analogously to the tri-layer model.

### 3.3 M3: stratified medium model

We compare the results from the multi layer modelling with a stratified medium analysis, which models the skin as a stack of multiple thin layers of varying dielectric function. From this model, the water gradient distribution within the SC and part of the epidermis is calculated [27]. For the optimization of the stratified model, an initial water distribution profile has been chosen similar to that proposed in [27] and [29]. More details about how we use this model with our THz hand-held probe and evaluate its usage for determining the skin hydration and thickness are given in our recent work [21].

### 3.4 Calculation of the skin hydration and SC thickness

In all three models detailed above we are building a multilayered system (either 3-layers, 4-layers or stratified with many layers). The properties of each layer depend on its dielectric function which is a function of the hydration (by means of the effective medium theory). The reflectivity, R , is related to the complex reflection coefficient r such that:

(3) $$R=|r{|}^{2}$$

By optimising the hydration and thickness parameters in the model to obtain the closest match to the experimental data, the properties of the skin are determined. Since the raw data acquired by our system are in the time domain, they are first Fourier transformed and then used to calculate the frequency dependent reflectivity, which can then be fitted to each theoretical model.

In all three models, the water concentration in the SC was calculated as a percentage based on the volume fraction from the measured permittivity through effective medium theory [30], which models the skin tissue as a binary composite system of water content and dry biological background (n=1.2 [27]). A value of 100 % would mean that the SC was composed entirely of water with no other components. In practice the maximum value for the SC hydration is typically 64 % [31].

## 4. Experimental results

**Fig. 3.**
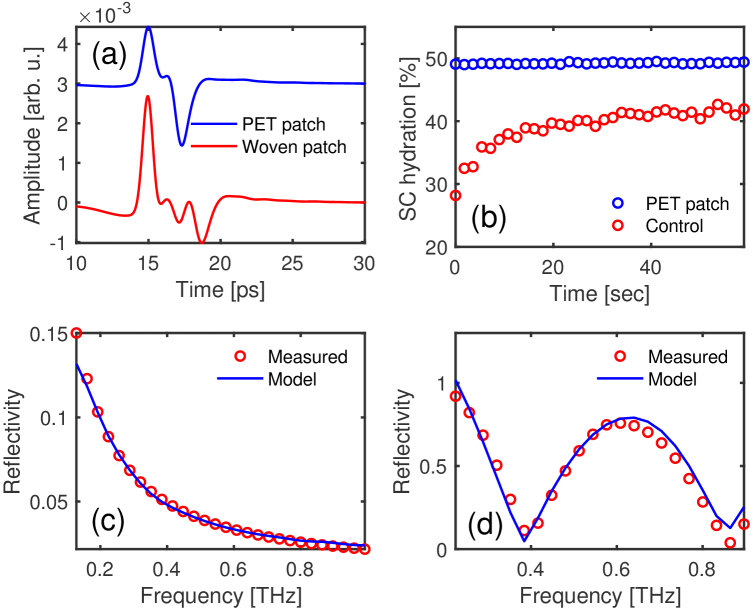
(a) Impulse response functions reflected from the PET and woven patch. (b) SC hydration percentage as function of time with the PET patch on (M2) and without the patch on (control, M1). (c) Experimental reflectivity of the control area (circles) and the fitted tri-layer model (M1) (line). (d) Experimental (circles) and theoretical (line) reflectivity of the PET patch on the skin using the four-layer model (M2).

After measuring the thickness of the patches (PET patch= 270μm±5μm , woven patch= 375μm±5μm ) and the dielectric properties of the patches shown in the previous section, the refractive index and thickness of the SC were calculated using the models detailed in Section 3. For clarity, we will label each sub-figure with M1, M2 or M3 to indicate which model was used to calculate the results presented therein. Throughout this section, for figures that are not plotting a result as a function of occlusion time, the results presented (for example, the SC hydration or SC thickness) are calculated from the data at 55s into occlusion. To account for system fluctuations and filter out noise, we used the deconvolution process in [18] to find the impulse response function from the measured data. Fig. 3 (a) shows the impulse response function reflected from the PET and woven patches for one of the volunteers. It can be observed from the figure that there are two reflected peaks from the PET patch, one from quartz-patch interface at 15 ps and another from patch-skin interface at 17 ps. While for the woven patch there are multiple internal reflections due to a more complicated structure of the patch, the pulse reflected from skin can still be easily detected at 18 ps. By fitting the tri- and four-layer models to the experimental data in the frequency domain, the water content of the SC, which is highly correlated with the refractive index through the effective medium theory, and its thickness can be found whether measured with or without the patches on the skin. The SC hydration levels for one of the volunteers after wearing the F1 PET patch and a control area C1 are shown in Fig 3 (b). This figure indicates that wearing the film patch occludes the skin so that its hydration is already saturated close to 50 % before the 1 min measurement, whilst the control area (without patch application) increased in hydration level from 28 % to approximately 40 %. This increasing trend in water concentration for the control area is because the skin is not able to exchange water with the environment when in contact with the quartz window and so water accumulates in the SC during the measurement. As depicted in Fig. 3(c) and (d), the addition of the patch changes the resulting reflectivity significantly. This is because it is made from very different material from skin.

**Fig. 4.**
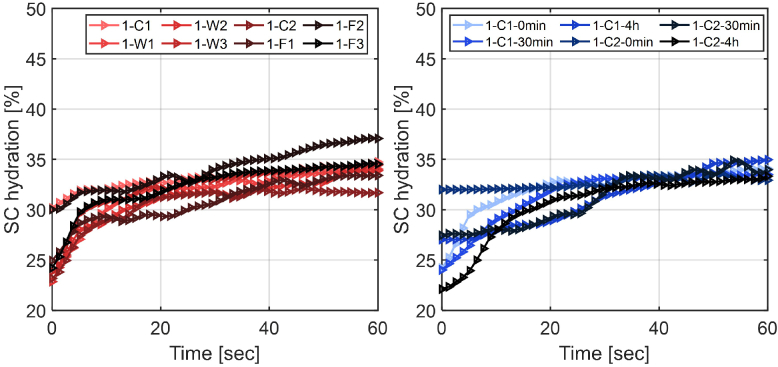
(a) SC hydration of the control areas (C1 and C2) and areas marked as F1, F2, F3, W1, W2 and W3 before the application of the patches (M1). (b) SC hydration of the control areas (C1 and C2) at 0min, 30min and 4hrs after removing the patches from the treated areas. These areas are measured for volunteer 1 (M1).

This process was repeated for all patches and all ten subjects in order to have a better understanding of the hydration levels. M1 was used to calculate the hydration at each of the eight areas as plotted in Fig. 4(a) for volunteer 1 before any of the patches were applied. Figure 4(b) shows the hydration for the two control areas at the subsequent times they were measured. We see that there is not much variation in the results for the different locations and also that the repeat measurements of the same control area are consistent. The hydration typically increases slightly with time due to the occlusion effect and so when comparing hydration values of the skin across volunteers and patches we use the value at 55s into occlusion where the change due to occlusion has levelled off. The fluctuations in the occlusion curve with time for some measurements are likely to be due to changes in the contact pressure.

**Fig. 5.**
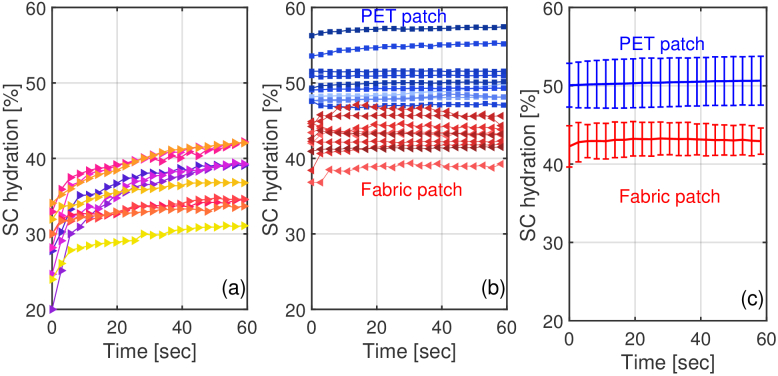
(a) SC hydration levels of the control area C1 for the ten volunteers as function of immediately after patch removal (M1). (b) SC hydration levels of the F1 and W1 areas for the ten volunteers (M1). (c) Mean values and standard deviation of (b) for the PET patch F1 and woven patch W1.

Figure 5 shows all the results acquired from the F1, W1 and C1 zones of ten subjects immediately after patch removal. It is evident from Fig. 5 (a) that for all the control zones the hydration levels increase as a function of time as a result of the occlusion of skin during the measurement. Meanwhile in Fig. 5 (b), for skin with a PET patch (F1) the hydration levels stay at a steady value, sometimes as high as 58 %, as wearing the PET patch has already caused water to significantly accumulate in the SC. In contrast, the woven patch (W1) only causes the skin to reach hydration levels lower than 50 %. This experiment demonstrates the occlusion capabilities of these two types of patches and shows that the PET patch is significantly more occlusive than the woven patch. This conclusion agrees with our assumption that the high porosity of the woven patches allows the skin to exchange more water with the environment. Finally, in Fig. 5 (c) mean values of the hydration levels of the ten volunteers for both PET and woven patches as well as the standard deviation are presented, clearly demonstrating the highly occlusive nature of the PET patch.

**Fig. 6.**
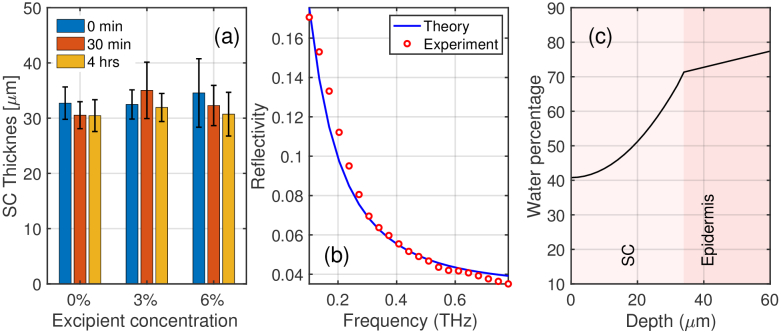
(a) SC thickness versus excipient concentrations for the PET (film) patches at 0 mins, 30 mins and 4 hrs after patch removal (M1). The error bars represent the standard deviation across the 10 volunteers. (b) Tri-layer model (M1) of reflectivity fitted to the experimental data. (c) Example water distribution within the skin using a stratified model (M3).

To further investigate the effects of the patches on the skin, all skin areas were also measured 30 min and 4 hrs after patch removal to study the recovery process. The SC thickness of the ten participants at different times after the application of PET patches is presented in Fig. 6. In Fig. 6 (a), the bars correspond to the average of the SC thickness at different times for the PET patches at different excipient concentrations, while the error bars indicate the standard deviation. Fig. 6 (b) is a typical tri-layer fitting showing the agreement between the theoretical and the experimental results in the frequency range of 0.1 THz to 0.9 THz. Fig. 6 (c) shows the resulting water gradient distribution from applying the stratified medium analysis to the skin [27].

**Fig. 7.**
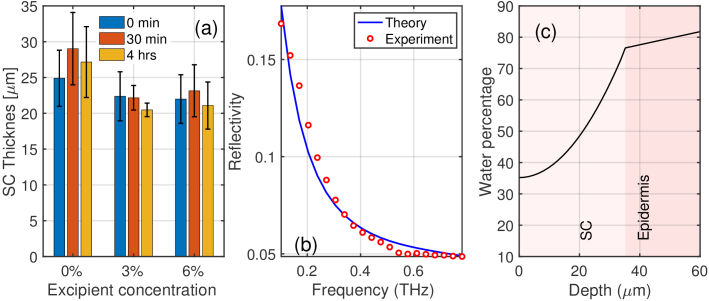
(a) SC thickness versus excipient concentrations for the woven patches at 0 mins, 30 mins and 4 hrs after patch removal (M1). The error bars represent the standard deviation across the 10 volunteers. (b) Tri-layers (M1) fitting to the experimental data. (c) Example water distribution within the skin using the stratified model (M3).

Figure 7 presents the same results as Fig. 6 but for the woven patches. In comparison, the application of PET patches has a stronger impact on the water distribution and thickness of SC: the SC thickness has an increase of approximately 10μm more than from the application of woven patches. This result further demonstrates the stronger effect film patches have on skin compared to woven ones.

**Fig. 8.**
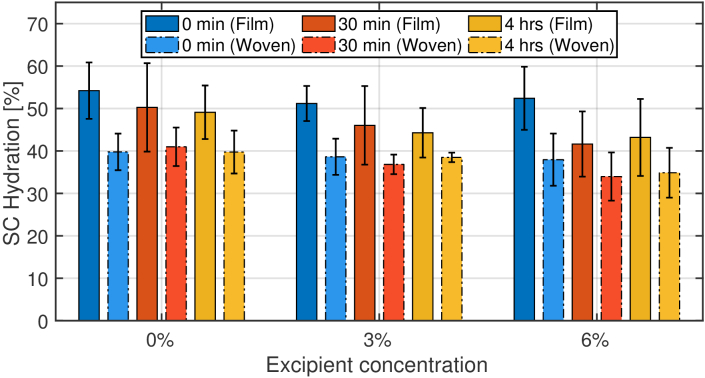
SC hydration content (at depth =0) at 0 mins, 30 mins and 4 hrs after removal of the PET patches (solid bars) and woven patches (dashed bars) versus the excipient concentration calculated from the stratified model (M3). The error bars correspond to the standard deviation of the ten volunteers.

Finally, the SC surface hydration (at depth = 0) after application of PET and woven patches with three excipient concentrations is shown in Fig. 8. The bars correspond to the extracted mean value after fitting the stratified model (M3) to the measured data while the error bars correspond to the standard deviation of ten applicants. These results suggest that the application of PET patches increases the hydration level to around 50 % at 0 min after peeling them off, which is 10 % higher than the hydration change induced by the application of woven patches. The hydration decreases with time after removing the patches showing that the skin is gradually recovering to its initial state. It is noteworthy that the skin takes longer than 4 hours to recover to its initial hydration after removing the patch, this is likely due to the skin being kept at a high hydration for an extended period of time (24hrs). Table 1 and Table 2 summarise the effects the excipient concentrations have on the SC hydration and SC thickness respectively. The error is the standard deviation of the 10 volunteers and this is relatively large due to person-to-person variation: some volunteers had drier skin than others. The values in Table 1 and Table 2 are calculated using the stratified model (M3) and the hydration tabulated is the mean across the 10 volunteers of the value at the outer surface of the SC (i.e. depth=0 in Fig. 7(c)). The hydration values show good agreement with the values from the tri-layer model in most cases. There are some small discrepancies as in M1 we assume a constant hydration value across the layer, whereas in M3 we are modelling the water profile as a curve.

**Table 1.** Mean SC hydration after the application of the film and woven patch (M3). The errors correspond to the standard deviation of ten volunteers.

Stratum Corneum Hydration [%]						
	Film			Woven		
Excipient	0 min	30 min	4 hrs	0 min	30 min	4 hrs
0 %	54.2 ± 6.6	50.2 ± 9.4	49.1 ± 6.3	40.8 ± 5.4	35.3 ± 3.5	34.6 ± 5.3
3 %	51.1 ± 4.1	46.0 ± 9.2	44.2 ± 5.8	39.3 ± 5.3	38.5 ± 2.4	38.8 ± 1.5
6 %	52.4 ± 7.4	41.6 ± 7.6	43.1 ± 9.0	39.9 ± 7.3	33.8 ± 6.9	34.9 ± 5.8

**Table 2.** SC thickness after the application of the film and woven patch (M3). The error bars correspond to the standard deviation of ten volunteers.

Stratum Corneum Thickness [ μ m]						
	Film			Woven		
Excipient	0 min	30 min	4 hrs	0 min	30 min	4 hrs
0 %	32.7 ± 2.9	30.5 ± 2.4	30.4 ± 2.8	24.8 ± 3.9	29.0 ± 5.0	27.1 ± 4.9
3 %	32.4 ± 2.6	35.0 ± 5.1	31.9 ± 2.5	22.3 ± 3.4	22.1 ± 1.7	20.4 ± 0.9
6 %	34.5 ± 6.2	32.3 ± 3.6	30.7 ± 3.9	21.9 ± 3.3	23.1 ± 3.6	21.0 ± 3.2

The hydration values of the skin after patch removal for the woven patches with excipient concentrations of 0 % and 3 % are all around 40 % which is fairly close to the mean starting value of around 33 %. This is because the woven patch allows the skin to exchange moisture with air well and so the skin hydration has not changed much from wearing the patch and so doesn’t change much during the recovery time. In contrast, the PET patch causes a significant increase in hydration for all excipient concentrations and does not return to its initial state within the 4 hours. It is interesting that the patches with 6 % excipient concentrations affect the skin such that it tries to over compensate after removal – for both the woven and PET patches, the hydration values dip slightly at the 30 minute post removal point. This warrants further investigation at more time points in future studies.

## 5. Conclusions

Water content and thickness of the SC have been studied on ten healthy volunteers after the application of six different types of patches commonly used for transdermal drug delivery treatment on the upper arm. Two types of backing materials for the patches (PET and woven) were used in this work and propylene glycol at three different concentrations (0 %, 3 % and 6 %) was added to the patches. Fitting of experimental THz measurements with a multi-layer optical model (M3) suggested that PET patches can occlude the SC and increase its hydration up to a level of 60 % after 24 hrs of application in contrast with woven patches which only increased the hydration level to around 40 %. This is consistent with our expectations considering the nature of the backing materials, as woven patches are not as occlusive as PET.

Occlusion by the quartz window on the control skin caused the SC hydration to increase quickly by more than 10 % during the 60s measurement for most volunteers, and the skin was able to recover back to its original hydration before the next measurement – typically taking 10 minutes for the SC to recover from a 60s measurement. However, for the skin treated with the patches, the skin took longer than 4 hours to recover to its initial hydration after removing the patch, likely due to the skin being kept at a high hydration for an extended period of time (24hrs).

We were also able to probe the swelling of the SC due to wearing the patches. The SC thickness calculated from our 3-layer model (M1) immediately after removing the patches was around 33μm for the PET patches, and around 25μm for the woven patches, the latter is closer to the SC thickness for the untreated (control) zones of around 20μm . Thus, the woven patches did not affect the SC thickness as much as the PET patches.

Measuring the rate and level of skin hydration changes caused by wearing TDD patches is a useful instrumental risk assessment tool. If a patch over-hydrates the skin quickly and for a long period of time, it indicates that the toxicological risk assessment of the patch components used must be performed carefully. At the moment, irritation and sensitisation experiments are carried out on animals. Apart from ethical considerations, such studies are expensive and long. The THz measurement of hydration levels can be an alternative to this and could be done non-invasively on humans over a shorter time period (e.g. potentially within 1 hour of wearing the patch). In short, these results demonstrate how THz spectroscopic analysis can be used as a powerful tool to study the evolution of SC thickness and skin hydration. This potentially is very useful, for example, for evaluating the performance of TDD methods in a non-invasive fashion.

## Data Availability

The data presented in this article are publicly available on Figshare at [32].
